# Elevated midbrain serotonin transporter availability in mixed mania: a case report

**DOI:** 10.1186/1471-244X-4-27

**Published:** 2004-09-13

**Authors:** Tommi Tolmunen, Mikko Joensuu, Pirjo Irmeli Saarinen, Hanna Mussalo, Pasi Ahola, Ritva Vanninen, Jyrki Kuikka, Jari Tiihonen, Johannes Lehtonen

**Affiliations:** 1 Department of Psychiatry, Kuopio University Hospital, 70210 Kuopio, Finland; 2 Department of Clinical Physiology and Nuclear Medicine, Kuopio University Hospital, 70210 Kuopio, Finland; 3 Department of Clinical Radiology, Kuopio University Hospital, 70210 Kuopio, Finland; 4 Department of Forensic Psychiatry, University of Kuopio, 70240 Kuopio, Finland

## Abstract

**Background:**

Results obtained from brain imaging studies indicate that serotonin transporter (SERT) and dopamine transporter (DAT) densities are altered in major depression. However, no such studies have been published on current mania or hypomania.

**Case presentation:**

In this single photon emission computed tomography (SPECT) study with [^123^I]nor-β-CIT we present a case with simultaneous symptoms of major depression and hypomania. She had an elevated serotonin transporter availability (SERT) in the midbrain and elevated dopamine transporter availability (DAT) in the striatum, which normalised in a one-year follow-up period during which she received eight months of psychodynamic psychotherapy.

**Conclusions:**

To our knowledge, this is the first report on SERT and DAT associated with mania. In our case the availability of both SERT in the midbrain and DAT in the striatum were elevated at baseline and declined during psychotherapy, while the SERT and DAT of the depressed controls increased during psychotherapy. Symptoms of hypomania in the case were alleviated during psychotherapy. Clinical recovery was also reflected in the Hamilton Depression Rating Scale (HDRS) scores.

## Background

Decreased serotonin transporter densities (SERT) have been recorded from the brains of depressed patients [1,2], which have also been reported to normalise during psychotherapy [3,4]. A few studies have also revealed altered levels of dopamine transmission in major depression [5,6]. However, in a search of the PubMed database we found no serotonin-specific brain imaging studies on mania or hypomania.

In this paper we present a case with simultaneous symptoms of major depression and hypomania. The structural diagnostic classifications DSM-IV and ICD-10 are unable to diagnose this kind of mixed state, even though similar cases have been presented earlier in the literature [7].

## Materials and methods

### Case

The index subject was a 25-year-old female with a history of two periods of major depression according to the DSM-IV-R criteria. She had also had hypomanic periods that did not completely fulfil the criteria of mania, and at baseline she fulfilled the criteria of hypomania, moderate depression and dysthymia (296.32, 300.40). She was overactive, restless and irritated and coped with a reduced amount of sleep. She had difficulty in concentrating on one thing at a time, and she appeared very lively and talkative. She was therefore diagnosed with bipolar mood disorder type II. In other words, our index patient had a mixed mania without fulfilling the criteria of full-blown mania during the baseline scan.

### Controls

The 6 female depressed controls were diagnosed to have severe or moderate depression (296.22, 296.23, 296.32 or 296.33). Three of them also fulfilled the criteria for dysthymia (300.40) and three had previously suffered periods of major depression. They were participants of our larger study on the effects of psychotherapy on SERT and DAT densities to be reported in full elsewhere.

The group of 10 healthy controls consisted mainly of employees of Kuopio University Hospital and medical students. Depression was an exclusion criterion for the control group. Controls had previously received no psychotropic medication or other psychiatric treatment and were physically healthy.

### Psychiatric evaluation

Psychiatric diagnosis was based on clinical assessment and verified for all study subjects by a trained independent psychiatrist using the Structured Clinical Interview for DSM-IV-R (SCID-I) [8]. The severity of depression was assessed with the 17-item Hamilton Depression Rating Scale (HDRS) [9].

### Setting, therapist and therapy

The depressed controls (see Table for sociodemographic characteristics) received psychotherapy for 12 months in an outpatient clinic of the Department of Psychiatry of Kuopio University Hospital. The case discontinued the therapy after eight months because she felt it was no longer useful to her. The case and the depressed controls had received no psychiatric treatment or medication prior the SPECT imaging and they were treated without medication. Psychotherapy sessions occurred twice a week, 80 sessions per year. The psychotherapists had formal postgraduate professional training in psychodynamic psychotherapy. The study design was approved by the ethics committee of Kuopio University Hospital.

**Table 1:** Characteristics of the participants. Values of the controls are means ± SD.

	Case	Depressed controls n = 6	Healthy controls n = 10
Age, y	25	27.2 (5.9)	26.3 (5.9)
Duration of depressive episode, months	9	10.6 (6.9)	-
SERT availability in midbrain at baseline	1.51	1.08 (0.12)	1.28 (0.12)
SERT availability in midbrain at 12 months	1.36	1.23 (0.20)	-
DAT availability in striatum at baseline	2.75	2.48 (0.35)	2.45 (0.25)
DAT availability in striatum at 12 months	2.61	2.70 (0.45)	-
HDRS score at baseline	15	17.67 (3.44)	-
HDRS score at 12 months	5	12.66 (5.09)	-

Abbreviations: DAT: Dopamine transporter, HDRS: Hamilton Depression Rating Scale SD: Standard deviation, SERT: Serotonin transporter.

Participants of the study were right-handed and all were female. The depressed and the healthy controls were age-matched with the case (Table). The healthy controls were not followed up.

### Imaging procedure

SPECT imaging was performed on the Wednesday after the menstrual bleeding that preceded the psychotherapy and on 12-month follow-up. A dose of 185 MBq of [^123^I] nor-β-CIT (supplied by MAP Medical Technologies OY, Tikkakoski Finland) was diluted in a volume of 10 ml physiological saline. The specific activity was higher than 1.8 × 10^11 ^Bq/μmol [10]. The dose was slowly injected into the right antecubital vein in a dimly lit and quiet room. Serial SPECT scans (5 min, 6 h and 24 h) were performed on a dedicated Siemens MultiSPECT 3 gamma camera with fan-beam collimators (Siemens Medical Systems; Hoffman Estates I11., USA) [11]. Head positioning was monitored during acquisition by using two position lasers.

### Data analysis

The SPECT scans were decay-corrected and reconstructed with Butterworth-filtered back-projection in a 128 × 128 matrix with a pixel size of 3 × 3 mm, and were attenuation-corrected with Chang's algorithm [10,11]. The imaging resolution was 8–9 mm. The SPECT slices were consecutively summarised to the slice thickness of 6 mm and re-aligned using a Siemens semi-automatic brain quantification program and the Talairach coordinates [12]. The slices were rotated and re-aligned so that transaxial (x-direction), sagittal (y-direction) and coronal (z-direction) slices were at right angles to each other.

Region of interest placement was based on a Siemens semi-automatic brain quantification program. The lower threshold of 60% of the maximum count was used to reduce the volume averaging and partial volume errors. Regions of interest were the cerebellum, striatum and the midbrain. It was assumed that the cerebellum (reference region) corresponds to a two-compartment model (unbound tracer in arterial blood and free plus non-specifically bound tracer in the tissue) [13]. The specific binding in ml/ml for SERT and DAT was calculated using a graphical plot [13]. The slope of this plot is equal to the distribution volume ratio: (Region - Cerebellum)/Cerebellum = V_D _- 1. The striatal uptake was pooled.

Functional neuroanatomy by means of SPECT was confirmed using magnetic resonance imaging (MRI) within two weeks of the SPECT imaging. If any cerebral focal abnormalities or organic brain diseases were detected by MRI scan, the patient was excluded from our study. The participants of our study thus had normal findings.

### Reproducibility of SPECT

The reproducibility of the SPECT scan had been previously studied with eleven healthy subjects (5 males and 6 females; age range 20–39 y). SPECT studies were performed twice with a 12-month interval. The correspondence between the studies was good, with the mean difference being 0.00 ± 0.08 (SD = standard deviation) for SERT (mean and SD: 1.27 ± 0.11 and 1.27 ± 0.14, respectively) and 0.04 ± 0.18 for DAT (mean and SD: 2.49 ± 0.28 and 2.45 ± 0.27, respectively). The intraclass correlation coefficient was 0.82 (p < 0.01) and 0.79 (p < 0.01), respectively (unpublished data).

### Statistics

The Student's t-test was used to compare the depressed controls and the healthy controls and paired samples t-test to compare SERT densities of the depressed controls at baseline and on follow-up. A p-value of less than 0.05 was considered as the criterion for statistical significance.

## Results

The background characteristics of the case and the controls are presented in the table. The case and the depressed controls had HDRS scores of 14 or more.

The midbrain SERT availability did not correlate with HDRS scores in depressive controls either at baseline or on follow-up. Neither did the change in the HDRS score correlate with changes in SERT or DAT capacities under therapy.

**Figure 1:**
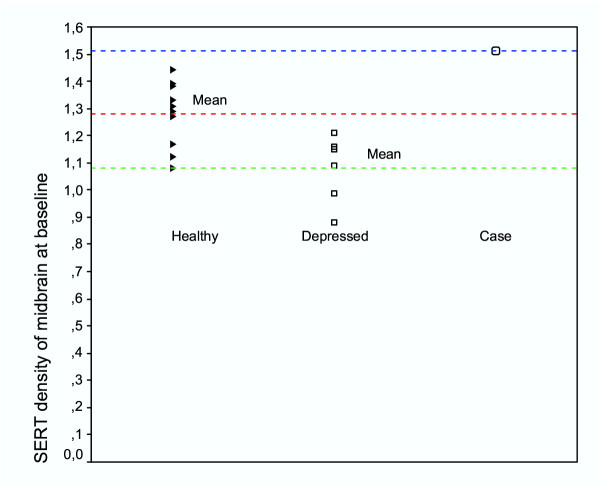
SERT densities of the healthy controls, depressed controls and of the index subject at baseline.

**Figure 2:**
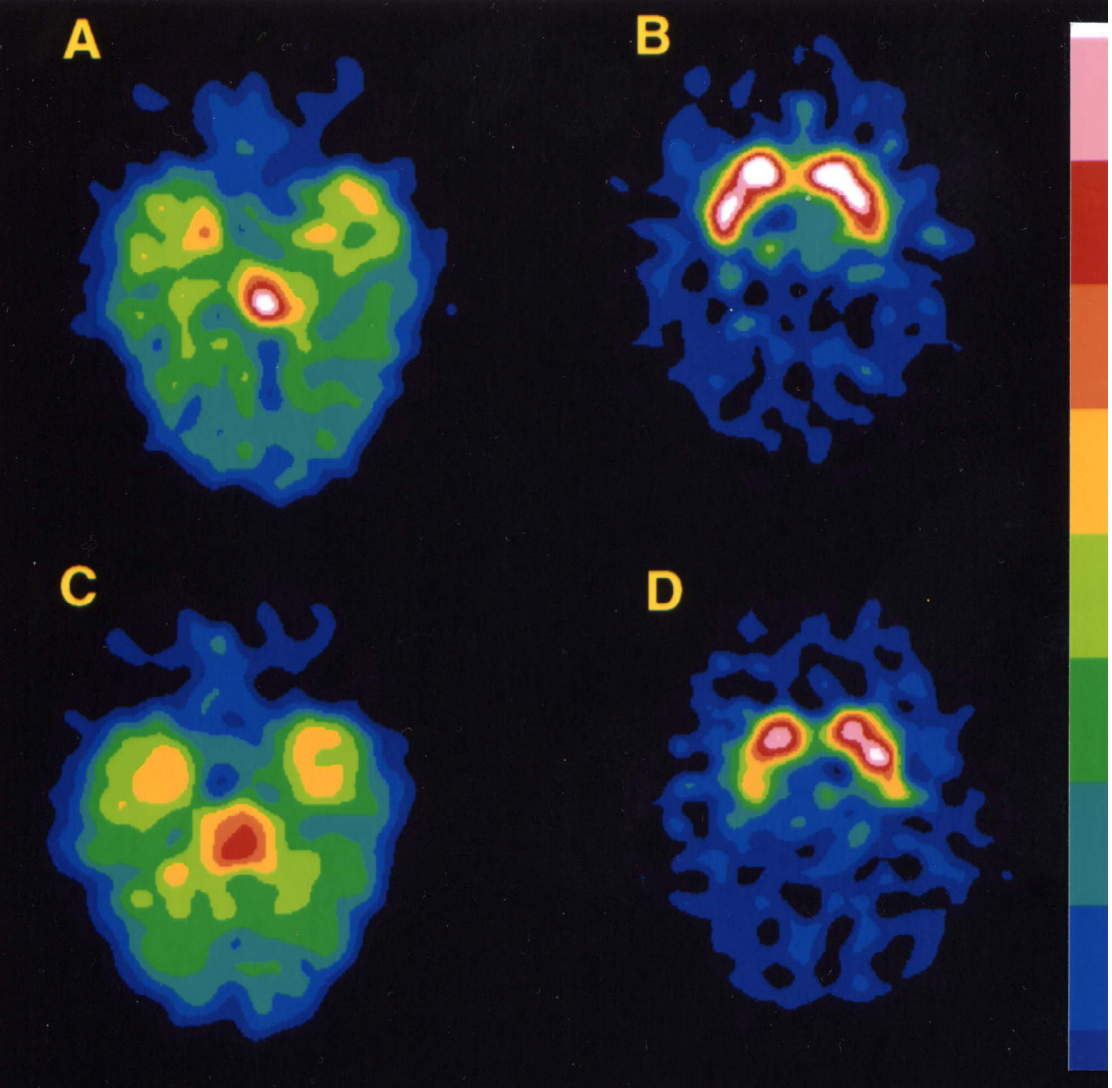
Transaxial slices of [^123^I]nor-β-CIT scans in the index subject at baseline (A and B) and after 12 months (C and D). A and C indicate midbrain SERT binding and B and D striatal DAT binding. A 10% step colour scale is shown on the right and the cerebellum was used in its normalization.

The case had an elevated SERT availability in the midbrain at baseline, while the depressed controls had decreased levels compared to the healthy controls (t = 3.17, p < 0.01; Table and Figure 1). The SERT availability of the index case was two standard deviations (SD) higher than the mean SERT availability of the depressed controls and almost two SD higher than the mean SERT availability of the healthy controls. The MRI scans of the case and the controls were normal. At the twelve-month follow-up, the HDRS scores of both the case and the depressed controls had decreased. The mean decrease in HDRS scores in depressed patients during the follow-up period was 5 (SD 3.3; t = 3.7, p = 0.02). The SERT availability in the midbrain had decreased in the case by 9.9% (Fig. 2) and increased in the depressed controls by 12.5% (t = 3.00, p = 0.03) during the one year of psychotherapy.

DAT densities in the striatum were elevated in the case (Table, Fig. 2.) and slightly elevated in the depressed controls at baseline compared to the healthy controls. On 12-month follow-up the DAT availability in the striatum of the case had decreased by 5.1%, while DAT densities in the depressed controls had increased by 8.8%. The increase in DAT binding was not significant (data not shown).

## Discussion

To our knowledge, this is the first report on SERT and DAT associated with a current mania. In our case the densities of both SERT in the midbrain and DAT in the striatum were elevated at baseline and they decreased during psychotherapy, while the SERT and DAT of the depressed controls increased during psychotherapy. Clinical recovery was also reflected in the change in the HDRS score. Furthermore, the index case had no symptoms of hypomania after the psychotherapy.

Ichimiya et al. [14] found increased SERT binding of the radioligand in the thalamus in a sample of patients with either major depression (n = 7) or bipolar disorder (n = 6) compared to healthy controls (n = 21). In their study the bipolar patients were either depressed or euthymic prior to brain imaging. There were no significant differences in binding potentials in the midbrain compared to the mood-disorder patients and the healthy controls. Bipolar patients had slightly higher binding potentials in the midbrain than the healthy controls, while patients with major depression had slightly lower binding potentials than the healthy controls. However, these differences were not significant. The patients of Ichimiya and co-workers were euthymic or depressed, so we cannot compare our results with theirs.

It is clinically well known that anti-depressive medication, including serotonin-selective drugs, may aggravate mania [15]. If the serotonergic functions had already been elevated in mania and had then been further enhanced by anti-depressive medication, it is reasonable that this might further increase the manic symptoms.

In animal models, the administration of an amino-acid mixture lacking the cathecolamine precursors tyrosine and phenylalanine decreases the availability of plasma tyrosine to the brain, which further diminishes cathecolamine synthesis [16]. In a few clinical human studies, decreasing tyrosine availability has had a positive effect on the symptoms of acute mania [17,18]. Tyrosine depletion has also led to decreased dopamine functions in healthy volunteers [19]. In positron emission tomography, a tyrosine-free amino-acid mixture increased striatal binding of the dopamine receptor ligand in healthy volunteers, which may reflect lowered presynaptic dopamine release [20]. SPECT studies revealing increased amphetamine-induced dopamine release in bipolar disorder patients have also been published [21]. The above-mentioned results indicate that acute mania may be associated with elevated dopaminergic functions in the central nervous system. A decrease in the DAT availability of the striatum was associated with clinical recovery in our case, which may support this hypothesis.

The DAT densities of the depressed controls were only slightly above the level of the healthy controls. Laasonen-Balk et al. [5] have previously observed a greater increase in DAT densities in depressed patients. Because of the small sample size of this study there was insufficient statistical power to draw any conclusions in this issue. Our results support previous findings that the SERT densities of depressed patients are lower than in healthy controls [1,2,4]. Previous studies on neurotransmitters in association with mania have mostly dealt with dopamine.

We cannot completely rule out the bias of nor-β-cit binding to the noradrenergic transporters (NORT). The noradrenergic cell-body rich nucleus ceruleus is close to our target region. This is assumed to contain the nucleus raphe, which is mostly comprised of serotonergic cell-bodies. The dopamine cell-body rich substantia nigra is also located close to our region of interest. However, nor-β-cit is considered to be more serotonin-specific than previous radioligands [10].

Three studies have been published on the association between SERT availability and the serotonin transporter genotype. Two of these were performed on healthy subjects [22,23], while the third concerned abstinent alcoholics and healthy controls [24]. In the study of Van Dyck and co-workers the short homozygotes had a significantly greater SERT availability than the long-short heterozygotes, which indicates a complex relationship between the genotype and SERT availability. Furlong et al. [25] found an association between promoter allele 2 of the SERT gene and bipolar disorder. Studies combining brain imaging and data of genotype are also recommended in the evaluation of bipolar disorder. PET would be a more valid method if adequate radioligands were available [14], as PET provides absolute rather than relative values for transporter availability.

## Conclusion

We have found no previous imaging studies showing that serotonin plays a role in mania. Further research is needed to determine whether our findings could be generalised to all manic states.

## Competing interests

None declared.

## Authors' contributions

TT planned the study and analysed and interpreted the data. MJ also interpreted the data. PIS initiated and planned the study and organised the psychotherapy setting. HM took care of the imaging procedures. PA organised the psychotherapy setting. RV was responsible for the MRI scans and the interpretation of the scans. JK performed SPECT analyses. JT participated in the design of the study. JL initiated and planned the study project. All authors read, critically revised and approved the final manuscript.

## Abbreviations

DAT: Dopamine transporter

HDRS: Hamilton Depression Rating Scale

SD: Standard deviation

SERT: Serotonin transporter

SPECT: Single photon emission computed tomography

## Pre-publication history

The pre-publication history for this paper can be accessed here:


